# A maturity model for the scientific review of clinical trial designs and their informativeness

**DOI:** 10.1186/s13063-024-08099-5

**Published:** 2024-04-19

**Authors:** S Dolley, T Norman, D McNair, D Hartman

**Affiliations:** 1 Open Global Health, 710 12th St South, Ste 2523, Arlington, VA 22202 USA; 2 The Bill & Melinda Gates Foundation, 500 Fifth Ave. North, Seattle, WA 98109 USA

**Keywords:** Clinical trial, Informativeness, Design review, Trial methods, Maturity model

## Abstract

**Background:**

Informativeness, in the context of clinical trials, defines whether a study’s results definitively answer its research questions with meaningful next steps. Many clinical trials end uninformatively. Clinical trial protocols are required to go through reviews in regulatory and ethical domains: areas that focus on specifics outside of trial design, biostatistics, and research methods. Private foundations and government funders rarely require focused scientific design reviews for these areas. There are no documented standards and processes, or even best practices, toward a capability for funders to perform scientific design reviews after their peer review process prior to a funding commitment.

**Main body:**

Considering the investment in and standardization of ethical and regulatory reviews, and the prevalence of studies never finishing or failing to provide definitive results, it may be that scientific reviews of trial designs with a focus on informativeness offer the best chance for improved outcomes and return-on-investment in clinical trials. A maturity model is a helpful tool for knowledge transfer to help grow capabilities in a new area or for those looking to perform a self-assessment in an existing area. Such a model is offered for scientific design reviews of clinical trial protocols. This maturity model includes 11 process areas and 5 maturity levels. Each of the 55 process area levels is populated with descriptions on a continuum toward an optimal state to improve trial protocols in the areas of risk of failure or uninformativeness.

**Conclusion:**

This tool allows for prescriptive guidance on next investments to improve attributes of post-funding reviews of trials, with a focus on informativeness. Traditional pre-funding peer review has limited capacity for trial design review, especially for detailed biostatistical and methodological review. Select non-industry funders have begun to explore or invest in post-funding review programs of grantee protocols, based on exemplars of such programs. Funders with a desire to meet fiduciary responsibilities and mission goals can use the described model to enhance efforts supporting trial participant commitment and faster cures.

**Supplementary Information:**

The online version contains supplementary material available at 10.1186/s13063-024-08099-5.

## Assessing quality in global health clinical trials

In addition to pharmaceutical industry (industry) funders, hundreds of global health clinical trials (CTs) are funded annually by private foundations, governments, and consortia. A meaningful number of these CTs end without being published or without trustworthy results [1–3]. A January 2024 query of ClinicalTrials.gov found 92 phase I–IV CTs currently active or enrolling participants that featured a majority of CT sites in sub-Saharan Africa. Industry—either alone or as leader of a funding group—funded 29.3% of the CTs; the US government funded 12.0% of CTs. The remaining 58.7% of CTs were funded by private foundations, with some contribution from other governments or organizations. These global health CTs had plans to enroll 91,200 participants (human research subjects). Before a CT begins, industry routinely performs scientific or methodological reviews on CT protocols to identify and address flaws in design. There is no direct evidence that other funders conduct such reviews. Because of this, it is imaginable that 70% of global health CT protocols do not receive a dedicated scientific review before enrolling their first study participants. This may account for the large difference in informativeness between industry and non-industry CTs found recently [4].

In its lifecycle, there are two phases prior to the CT’s start and participant recruitment. First is a phase when the CT has not procured a funding commitment (pre-funding), and then the second is a post-funding phase. The dominant approach used by government funders to decide if a research study will be funded is peer-review. While peer-review for pre-funding decisions is well established, it continues to evolve and not necessarily in a scientific direction. For example, a large fraction of stakeholders believe peer-review ought to change to only assess the investigator, not the proposed project, or include a lottery [5]. One systematic review found that, in pre-funding peer-review, comments on research design represented 2%, methodology 4%, and methodological details 5%, respectively, of total comments [6]. During pre-funding, these reviewers also needed to comment on dozens of other factors [6]. This dynamic—along with the sometimes-large time gap between pre-funding and CT inception and the design changes therein—makes peer review inadequate for scientific design review.

In the post-funding phase, there are two other types of review that focus on elements outside of CT design. These reviews and related concepts are described in Table 1. The two reviews that happen completely or primarily in post-funding and before participant recruitment begins are regulatory and ethical. The regulatory and ethics review domains are relatively mature and well-developed.

**Table 1:** Types of reviews for clinical trials

Review type	Definition
Pre-funding peer review	Researchers, academics, and scientists—recruited by funders to volunteer their efforts—assess applications from their peers that solicit funding for clinical studies. This evaluation, performed by experts external to the funder, is “used to decide whether studies will be funded” [7–9]
Regulatory review	“An investigation of the proposed CT protocol to assess whether the CT will expose subjects to unnecessary risks, qualifications of investigators, commitments to obtain informed consent, intent to both gain approval from an independent review board and comply with drug regulations” [10]. Alternately, “assessment and validation of the submitted regulatory documents, assessment of study protocol, scientific evidence to show the product is safe for the study, the risk and benefits of patients’ participation in the study; qualification of the study team, commitment to follow regulatory and Good Clinical Practice guidelines and to protect participants, and ensuring ethical clearance has been achieved.” (communication, Dr. Beno Yakubu, Nigeria Agency for Food & Drug Administration, April 16, 2022)
Ethical/bioethical review	Joint consideration by expert group members on a CT’s ability to follow best practices in seven areas: “respect for subjects, informed consent, independent review, favorable risk–benefit ratio, fair subject selection, social and clinical value, and scientific validity” [11]. “The process must assess risk–benefit in areas such as participant consent, confidentiality, data security, minimizing harms to participants; in return for patient risk, the study must conform to scientific principles, and take into account the existing body of evidence, and make a contribution to generalizable knowledge” [12–14]
Scientific design review	An evaluation focused on study protocol details that estimates and describes risks of not achieving statistically sound and meaningful results—such a review must evaluate biostatistical methods, question formulation, inclusion and exclusion criteria, commonality of endpoints, site selection, prudence of prevalence and effect assumptions, and more, without focus on generic good clinical practice, ethics, and regulatory topics that will be covered by others. A research unit wrote “[our] protocol review monitoring committee is the scientific review panel, responsible for ensuring the scientific merit and rigor of the protocol, while the Institutional Review Board ensures that the study is ethical and safe” [15, 16]

Ethical and regulatory reviews both overlap in limited ways with consideration of CT design methods. “It is clear that scientific assessments are a source of confusion for some ethics committees…ethics committee members revealed that they often had doubts about whether scientific validity is within their purview” [12]. Because the focus of an ethics review is *not* assessing optimal CT methods, “ethicists entering a review may be concerned about whether they have “the scientific literacy necessary to read and understand a protocol” [12]. Regulators and ethicists in low resource settings are often not trained in the scientific disciplines necessary to evaluate CT design risk—such as biostatistics and pharmacokinetics. Members of Institutional Review Boards seeking to deliver on their primary purpose—delivering an International Council for Harmonisation E6, E8, E9, and Good Clinical Practice guideline-supported participant protection review—and members of regulatory boards seeking to deliver on safety and participant protection may, justifiably, take only a secondary look at a CT’s statistical details. A cursory assessment of methods by an ethics committee may be necessary for them, but it may not be sufficient for funders. Likewise in the regulatory realm: the review of a protocol post-funding will include only targeted scientific assessment, since, for regulators, the focus on safety and similar matters crowds out efforts to identify more optimal approaches in CT design.

This state of affairs leaves an opportunity gap for scientific review of global health CT designs post-funding and prior to CT start. Industry performs scientific design reviews; it may or may not be coincidental that industry funded CTs were more likely to be informative during COVID than those CTs funded by others [17]. The US cancer academic CT community—funded by the US government—has created programs to comply with mandated post-funding scientific review of grantee CT designs. Multiple government and private CT funders, who to date have only performed pre-funding peer-reviews, are investigating the cost and effort involved with adding reviews of protocols. It is often only at the protocol stage of trial planning when a funder can see specifics such as whether the trial design is informed by systematic evidence; more advanced, pragmatic, or participant-centric design; or the presence of concrete recruitment plans, statistical analysis plans, or sample size simulations. As yet, standards do not exist.

## Informativeness

Informativeness is a characterization of a CT that indicates the study will achieve its recruitment, statistical power, and other design goals, resulting in credibly answering its research questions. An informative CT “provides robust clinical insight and a solid on-ramp to either the next phase of development, a policy change, a new standard of care, or the decision not to progress further” [18]. Uninformative results are widespread. One study found only 6% of CTs funded outside of industry met all four conditions for informativeness [4]. Across a number of stakeholders working to identify design practices associated with uninformativeness, there is consensus on a core set of failures. These include principal investigators (PIs) being unrealistic or overly optimistic in their ability to set and achieve feasible and appropriate sample sizes and non-use of evidence-based disease burden and effect rates [17, 19–21]. “Studies that failed to influence policy change or a confident next step in a go/no-go decision were associated with factors such as lack of use of common endpoints, lack of conservatism in effect estimates, not using biostatistical simulation to derive sample sizes, using unduly restrictive inclusion criteria, and avoiding use of innovative CT designs” [18]. Qualities that drive informativeness are almost all defined during the design phase of the CT. Eleven of Zarin et al.’s twelve “red flags” for uninformativeness can be identified before a CT begins recruiting [22]. A multi-stakeholder working group of experts led by the Experimental Cancer Medical Centres made recommendations on how to improve CTs. Seven of the group’s ten consensus recommendations could or must be planned and addressed during the design phase of a CT [23]. Because likelihood of informativeness is cemented from a PI’s design work and design choices, post-funding scientific design reviews have high potential to identify risks of uninformative outcomes and suggest fixes before the CT is finalized and cannot be changed.

## A maturity model for scientific design reviews of clinical trials

A maturity model is a helpful tool for knowledge transfer to help grow capabilities in a new area, or for those looking to perform a self-assessment in an existing area. Such a model is offered for scientific design reviews of CT protocols: given time and funding, a chance to identify opportunity gaps in CT design, analysis, and communication. This maturity model includes 11 process areas and 5 maturity levels. Each of the 55 process area levels is populated with descriptions on a continuum toward an optimal state to improve CT protocols in the areas of risk of failure or uninformativeness.

A maturity model is “a tool that helps assess the current effectiveness of a person or group and supports figuring out what capabilities they need to acquire next in order to improve their performance” [24]. As an organization desires to implement CT scientific design/methodology reviews, or improve existing reviews, a maturity model can help to improve quality and capacity.

There are a number of variants of maturity models. A suitable model for presenting a maturity model is the Object Management Group Business Process Maturity Model (BPMM-OMG) [25]. Maturity levels (ML) are displayed on the *Y*-axis and are “well-defined evolutionary plateaus toward achieving a mature…process” [26]. The ML titles specific to BPMM-OMG and their fixed definitions are shown in Table 2. These levels act as ratings or grades for parts of a review process.

**Table 2:** Maturity levels (BPMM-OMG)^a^

Maturity code	Maturity levels	Maturity level definition
ML5	Innovating	Wherein both proactive and opportunistic improvement actions seek innovations that can close gaps between the organization’s current capability and the capability required to achieve its business objectives
ML4	Predictable	Wherein the capabilities enabled by standard processes are exploited and provided back into work units. Process performance is managed statistically through the workflow to understand and control variation so that process outcomes can be predicted from intermediate states
ML3	Standardized	Wherein common, standard processes are synthesized from best practices identified in the work groups and tailoring guidelines are provided supporting different business needs. Standard processes provide an economy of scale and a foundation for learning from common measures and experience
ML2	Managed	Wherein management stabilizes the work within local work units to ensure that it can be performed in a repeatable way that satisfies the workgroup’s primary commitments. However, work units performing similar tasks may use different procedures
ML1	Initial	Wherein business processes are performed in inconsistent sometimes ad hoc ways with results that are difficult to predict

^a^Maturity level definitions here are taken directly from BPMM-OMG

Capabilities, as represented in maturity models, are often called process areas (PA). PAs are one or more grouped workstreams performed to meet a need [26]. To create a usable maturity model, users must carefully select the range of capacity and efforts—the cluster of related activities: in order to evaluate a scientific design review practice, the process areas must be identified and organized. At The Bill & Melinda Gates Foundation, after developing a post-funding scientific design review program across multiple disease areas and with multiple study types, eleven PAs were identified as independent capabilities key to the program. These PAs were curated by the authors after program progress through maturity levels, participation in all areas of the program, and non-systematic interviews with other program staff. These PA descriptions for scientific design reviews are shown in Table 3. In each “cell,” or capability cluster at a particular level of maturity, the contents include examples of mastery at that level. This comprehensive set offers a new or existing practitioner the benefit of including what matters and excluding what does not, resulting in time and cost savings, better CTs, and risk reduction.

**Table 3:** Process areas for performing scientific design reviews of clinical trials

Process area	Process area definition
Informativeness-centric	A scientific design review where the main focus is on identifying and reducing risks that the CT will end without definitively answering its research question. An informativeness-centric review leaves as secondary any design concerns tied exclusively to regulatory, bioethics, and clinical operations topics. Focus is on evidence-based drivers of informativeness, such as sample size methods, use of local up-to-date epidemiological data as input variables, conservative effect estimates, use of biostatistical simulation, and use of common endpoints
Breadth of review expertise	Every CT has a variety of attributes that might make it distinctive. These attributes may appear across a range of CT elements, such as the intervention, stage of the disease, CT phase, CT site(s), or design characteristics. Breadth of review expertise means the expert review panel includes, for most or all unique attributes, a reviewer who has implemented, provided oversight for, designed, or critiqued that attribute in the past. This represents how complete the application of reviewer expertise to all details of a CT can be. This is often correlated with more, rather than fewer, reviewer individuals on a panel
Depth of reviewer expertise	Depth of expertise means the review panel includes, for key attributes of a CT and its design, reviewers who have designed and implemented, participated in, or provided oversight for related CTs. The reviewer is known to others in the field as being a well-known or famous resource or author on intricacies, advanced methods, or the corpus of work in a specific topic; typically, this requires decades of experience
Iterative	There are multiple rounds of analysis, edits, and collaboration in the review. Each expert sub-panel or working group iterates its findings and recommendations. Sub-panels consolidate and submit their review to a higher-level panel, which iterates with the sub-panels and within itself. The higher-level panel iterates the review with the PI. The iterations ensure each critique and recommendation has been refined, prioritized, and understood
Information-enhanced	There is a wide variety of information beyond the protocol that could indicate the risk level and riskiest attributes of a CT’s design. This information, if curated, and put in the hands of reviewers, makes for a richer review. An information-enhanced review means one where the protocol is accompanied by information incremental to the protocol requested by reviewers and sourced from PI or internally that provides risk insights to the reviewer
Solution-oriented	Solution-oriented means reviews ought to focus on solutions to multiple stakeholders’—but especially PIs’—challenges as well as the challenges inherent in design attributes. The solutions offered ought to be specific, timely, feasible, and informativeness-forward. Solutions could include links to other experts, additional funding such as CT planning grants, data, or other resources
Software-enabled	Software-enabled scientific design review means all relevant portions of the process that can be reliably enhanced with technology would be. This ranges from basic mechanics such as scheduling, communication, and secure document sharing, all the way to the use of artificial intelligence for prediction and data mining. Software could be used to support other process areas, such as measuring time spans, or for scouring registries, databases, historical protocols, and publications toward information enhancement
Collaborative	A collaborative review process is one that is increasingly communicative within and across stakeholder groups. This communication and collaboration could be flexible enough to adjust to changes in context. Collaborations could range from enabling quick scheduling and correspondence to partnering more deeply in-person, telephonically, or with other real-time engagement. Reviewers speaking to PIs about protocol review findings and recommendations is a crux of collaboration
Rich in data and analytics	A review program rich in data and analytics is one that collects, cleans, curates, and enriches information about all parts of reviews and uses analysis and visualization to communicate more richly with stakeholders, answer questions, and aid in actionable decision-making, as well alerting to trends and finding opportunities
Reliability and quality	The platform and approaches to delivering reviews perform their intended function. Reviews and the mechanics of delivering them are dependable. The team and platform sustain a level of quality over time. There is an increasingly lower number of fails and defined approaches to fix failed reviews. Stakeholders perceive quality and value in reviews. There is a consistency of delivery over time; costs are maintainable
Time appropriate	The review approach considers time sensitivities of disease urgency and current context and needs of the funders, PIs, and other stakeholders. Each segment in the review process may consider, relative to different facets of time, the attributes of sustainability, routineness, elasticity, rigidity, or fragility. Organizations are precise around trade-offs related to timing and deliberate in their application of time aids and boundaries

Once a maturity model variant is selected and the topic-specific PAs are populated, users can plot the maturity levels for each PA. In the case of a maturity model for scientific design reviews, there are 11 PAs with 5 maturity levels each. All 11 PA tables in this maturity model are included in the supplementary material. The first PA table, support for CT informativeness, is reproduced here as an exemplar of the remaining PA tables (Table 4).

**Table 4:** Process area 1, informativeness-centric. An informative CT includes a hypothesis that addresses an important and unresolved scientific, medical, or policy question; is designed to provide meaningful evidence related to this question; must have a realistic plan for recruiting sufficient participants; must be conducted and analyzed in a scientifically valid manner; and reports methods and results accurately, completely, and promptly [27]. An alternate definition is that an informative CT is designed to have the best chance to complete on time, answer its research questions definitively, and effect policy change or a regulatory process, through special commitment to (a) siting the CT based on epidemiology and impact rather than convenience, (b) completing a statistical analysis plan concurrently with the CT protocol, (c) using accepted endpoints and conservative effect and prevalence/incidence estimates, and (d) utilizing contemporary techniques, such as statistical simulation, innovative CT designs, and software to monitor recruitment

**Maturity Code**	**Maturity Levels**	**Capabilities, Informativeness-centric review**
ML5	Innovating	Review organization is efficiently applying artificial intelligence, digital innovation, and novel data to each review to surface informativeness risk. New research, based on studies that end informatively, is being performed to identify true drivers of informativeness. Innovations are invested in that make drivers to informativeness more clearly identified by reviewers. Improvements to continuing education for reviewers have occurred. Uncovering new drivers to uninformativeness happens through convenings of peers, talks from visionaries and researchers, and other engagement. Methods of innovative, effective continuing education for expert reviewers who are not pure CT methodologists are explored. Because of the pace of technology improvement and new inventions in the space, not all innovations can be adequately captured and described here.
ML4	Predictable	Informativeness information in the public domain specific to the design to be reviewed is collected and provided to reviewers along with the review materials: review-specific ‘benchmarks’ of similar CTs crafted just in time. Statistics on historical coverage of informativeness review variables are collected and provided to reviewers to ensure more complete reviews. As each review progresses, at interim points early indicators of trending areas of concern amongst reviewers, and/or other-generated risk scores are provided to all reviewers.
ML3	Standardized	The review organization selects the most appropriate definition of informativeness. A list of common drivers of uninformativeness is made, referred to, and used to educate and orient reviewers. This list is sourced empirically and reinforced with clinical study exemplars. The primacy of informativeness over good clinical practice, regulatory, ethical, or other types of recommendations is mentioned often and debated. The large majority of review recommendations tie to informativeness rather than Good Clinical Practice, regulatory, reducing bias, or other areas of interest. All stakeholders beyond reviewers are prompted to understand informativeness and its drivers.
ML2	Managed	Across reviewers, there is a mix of education level on uninformativeness and what causes it. There is variation in review output based on varying levels of CT experience and other variables. Some reviews have documented recommendations with rich meta-information, others have limited context, others have no documented output other than a discussion. Informativeness is specified as the most important quality of a CT, and the purpose of the review only sometimes focuses on informativeness. Sometimes reviewers ignore the informativeness focus and default to their own focus preferences (regulatory, ethics, clinops, equity, other). Reviews may miss obvious PI shortcuts that increase risk of uninformativeness.
ML1	Initial	Focus on informativeness varies often. Many reviewers believe the word informativeness refers to the traditional definition, rather than being a term of art tied to CT design rigor. Review comments optimize the study design for items unrelated to informativeness, such as safety, ethics, or clinical operations. There is no clear, written statement that the review is about informativeness. There is no discussion or education about the fact that a CT could fail to be informative. PIs can avoid being reviewed if desired. Presence of specific reviewer roles decides whether certain informativeness levers are reviewed.

## Discussion

In 2020, The Bill & Melinda Gates Foundation developed and implemented an approach to performing post-funding scientific design reviews for CTs developed by its grantees. The review program, as it evolved, became more complex to support high quality reviews in large volume [28]. It is likely this program generates positive impact via reducing the risk of uninformativeness, through its non-mandatory, expert recommendations for protocol changes prior to trial start. The relevance for other CT funders is high, as uninformativeness seems an endemic problem. That said, the applicability of progressing to high maturity in the model presented may be low due to a perception of little time and resources among funders. Time and funding constraints also limit the ability of PIs to implement some expert recommendations [29]. Recommendations to a PI to add significant changes to a protocol—such as the addition of a systematic evidence to inform design, a clear element of informativeness—would need to be funded by a trial planning grant.

Many post-funding scientific design reviews happen globally outside of industry, although less frequent than pre-funding, pre-protocol peer reviews. The non-industry funders of protocol reviews—such as government-funded entities, private foundations, and the United States National Institutes of Health Cancer Center academic trial funders—operate at a variety of maturity levels. In such cases, those funders interested in improving or assessing their existing protocol review programs might consider using either the Maturity Model herein or a simplified version. For example, a funder wanting to add post-funding protocol review to their pre-existing pre-funding peer review might use the model herein but leave out process areas such as (a) having a wide breadth of expertise in a large reviewer team (PA2), (b) having within-review iterations (PA4), and (c) being software-enabled (PA7).

Adopting this maturity model for post-funding scientific design reviews has strengths and limitations. Strengths include (a) the model offers measurement, and an implied pathway toward maturity, in a variety of key areas—some necessary—for delivering scientific design reviews; (b) the model is focused on addressing risk in areas most likely to fail in CTs—trial informativeness; and (c) the model was developed, adjusted, and updated based on learnings from completion of over 100 protocol reviews. Limitations include (a) adopting a commitment to multi-element excellence within eleven process areas makes for a complicated model, (b) the expense involved in pursuing this approach may be challenging for some funders to take on, and (c) due to confidentiality requirements, the foundation is not able to provide detailed examples of its program in action.

## Conclusions

Industry-sponsored CTs were found to have, in select situations, significantly higher informativeness than private funder-sponsored CTs [4]. A large portion of global health CTs are supported by private funders. There is interest among private funders to adopt the multi-expert scientific design reviews in use by industry and select government and foundation funders. Peer-review of CTs today offers too little time for a rigorous evaluation of CT design and associated methods. Creating persistent improvement in a CT protocol is most likely achieved by implementing a scientific design review, and the best time for this is late in the design phase or close to when the protocol is finalized. The maturity model described can help funders who do not have an approach for creating a post-funding scientific design review program. If private funders do have such a program, this maturity model can help extend its depth and breadth. The model offers both a formative structure and a continuum promising improved precision, efficacy, collaboration, and communication. The benefit accrues to private and government funders, industry, CT participants, and global citizens alike through increased likelihood of CT informativeness and faster cures.

### Supplementary Information

**Additional file 1.** From A maturity model for the scientific review of clinical trial designs and their informativeness. **Table S1.** Process Area 1, Informativeness-centric. **Table S2.** Process Area 2, Breadth of review expertise. **Table S3.** Process Area 3, Depth of reviewer expertise. **Table S4.** Process Area 4, Iterative. **Table S5.** Process Area 5, Information-enhanced. **Table S6.** Process Area 6, Solution-oriented. **Table S7.** Process Area 7, Software-enabled. **Table S8.** Process Area 8, Collaborative. **Table S9.** Process Area 9, Rich in data & analytics. **Table S10.** Process Area 10, Reliability and quality. **Table S11.** Process Area 11, Time appropriate.

## Data Availability

The dataset analyzed during the current study is available in the ClinicalTrials.gov repository, found at https://clinicaltrials.gov/.

## Abbreviations

BPMM-OMG: Business process maturity model from Object Management Group
CT: Clinical trial
PA: Process area
PI: Principal investigator
