# Supplementary material for: A maturity model for the scientific review of clinical trial designs and their informativeness
Source: Trials. 2024 Apr 19;25:271. doi: 10.1186/s13063-024-08099-5 (PMC11027356; doi:10.1186/s13063-024-08099-5)
Supplement: Supplementary file 1 — Additional file 1. From A maturity model for the scientific review of clinical trial designs and their informativeness. Table S1. Process Area 1, Informativeness-centric. Table S2. Process Area 2, Breadth of review expertise. Table S3. Process Area 3, Depth of reviewer expertise. Table S4. Process Area 4, Iterative. Table S5. Process Area 5, Information-enhanced. Table S6. Process Area 6, Solution-oriented. Table S7. Process Area 7, Software-enabled. Table S8. Process Area 8, Collaborative. Table S9. Process Area 9, Rich in data & analytics. Table S10. Process Area 10, Reliability and quality. Table S11. Process Area 11, Time appropriate. [file 13063_2024_8099_MOESM1_ESM.docx]

**Supplement 1, from A maturity model for the scientific review of clinical trial designs and their informativeness**

**Supplementary Material**

The following pages include, if needed, definitions or reference information prior to the process area-specific table of maturity model capability and performance descriptions.

Process Area 1, Informativeness-centric

An informative trial includes a hypothesis that addresses an important and unresolved scientific, medical,

or policy question; is designed to provide meaningful evidence related to this question; must have a realistic plan for recruiting sufficient participants; must be conducted and analyzed in a scientifically valid manner; and reports methods and results accurately, completely, and promptly. [1] An alternate definition is that an informative trial is designed to have the best chance to complete on time, answer its research questions definitively, and effect policy change or a regulatory process, through special commitment to a) siting the trial based on epidemiology and impact rather than convenience, b) completing a statistical analysis plan concurrently with the trial protocol, c) using accepted endpoints and conservative effect and prevalence/incidence estimates, and d) utilizing contemporary techniques, such as statistical simulation, innovative trial designs, and software to monitor recruitment.

**Table S1. Process Area 1, Informativeness-centric**

| **Maturity Code** | **Maturity Levels** | **Capabilities, Informativeness-centric review** |
| --- | --- | --- |
| ML5 | Innovating | Review organization is efficiently applying artificial intelligence, digital innovation, and novel data to each review to surface informativeness risk. New research, based on studies that end informatively, is being performed to identify true drivers of informativeness. Innovations are invested in that make drivers to informativeness more clearly identified by reviewers. Improvements to continuing education for reviewers occur. Uncovering new drivers to uninformativeness happens through convenings of peers, talks from visionaries and researchers, and other engagement. Methods of innovative, effective continuing education for expert reviewers who are not pure trial methodologists are explored. Because of the pace of technology improvement and new inventions in the space, not all innovations can be adequately captured and described here. |
| ML4 | Predictable | Informativeness information in the public domain specific to the design to be reviewed is collected and provided to reviewers along with the review materials: review-specific ‘benchmarks’ of similar trials crafted just in time. Statistics on historical coverage of informativeness review variables are collected and provided to reviewers to ensure more complete reviews. As each review progresses, at interim points early indicators of trending areas of concern amongst reviewers, and/or other-generated risk scores are provided to all reviewers. |
| ML3 | Standardized | The review organization selects the most appropriate definition of informativeness. A list of common drivers of uninformativeness is made, referred to, and used to educate and orient reviewers. This list is sourced empirically and reinforced with clinical study exemplars. The primacy of informativeness over good clinical practice, regulatory, ethical, or other types of recommendations is mentioned often and debated. The large majority of review recommendations tie to informativeness rather than GCP, regulatory, reducing bias, or other areas of interest. All stakeholders beyond reviewers are prompted to understand informativeness and its drivers. |
| ML2 | Managed | Across reviewers, there is a mix of education level on uninformativeness and what causes it. There is variation in review output based on varying levels of trial experience and other variables. Some reviews have documented recommendations with rich meta-information, others have limited context, others have no documented output other than a discussion. Informativeness is specified as the most important quality of a trial, and the purpose of the review only sometimes focuses on informativeness. Sometimes reviewers ignore the informativeness focus and default to their own focus preferences (regulatory, ethics, clinops, equity, other). Reviews may miss obvious PI shortcuts that increase risk of uninformativeness. |
| ML1 | Initial | Focus on informativeness varies often. Many reviewers believe the word informativeness refers to the traditional definition, rather than being a term of art tied to trial design rigor. Review comments optimize the study design for items unrelated to informativeness, such as safety, ethics, or clinical operations (clinops). There is no clear, written statement that the review is about informativeness. There is no discussion or education about the fact that a trial could fail to be informative. PIs can avoid being reviewed if desired. Presence of specific reviewer roles decides whether certain informativeness levers are reviewed. |

Process Area 2, Breadth of reviewer expertise

**Table S2. Process Area 2, Breadth of review expertise**

| **Maturity Code** | **Maturity Levels** | **Capabilities, Breadth of review expertise** |
| --- | --- | --- |
| ML5 | Innovating | Unique approaches are developed to gain expertise when an expert cannot be sourced. For example, digital mining of prior art and summation through natural language processing, or pre-automated blitz surveys of a relevant PI community at large, could assemble expertise. Artificial-intelligence-powered recruiting techniques could find new experts. Automated identification of best practices in a range of expert areas from mining protocols, trials, and past reviews could be applied in lieu of a human. Because of the pace of technology improvement and new inventions in the space, not all innovations can be adequately captured and described here. |
| ML4 | Predictable | Experience with spikes in review demand informs when to build in redundancy of SMEs, curators, and managers, to have multiple review teams available with full breadth of review expertise. Insight into the ability of doing run-time recruiting of expertise beyond the breadth of the current portfolio of experts is known. Approaches exist for how to find, stage, and staff expertise for rare or complex interventions, diseases, and trial types when they map to specific review timelines, and requests are all predictable and routinized. Information on trial sites most commonly reviewed is used to identify the ideal geographic background of experts from low-resource settings. Data exists to present the elasticity of value of expertise breadth, toward understanding how many experts in diverse but applicable niches is the right amount per review. |
| ML3 | Standardized | A core set of minimum expertise coverage across expert roles is set and is attained consistently, or the review is not performed. The 'mix' of experts on a single review team and the relative ratio of headcount is established. Some policy around run-time requests from stakeholders for specific expertise is set. Policies on funding expert reviewers, ranges of rates, and levels of effort and definition of scope within segments of a review are established. Business rules exist to know when a review team will include an expert for the trial site geographies; an expert specializing in gender equity or other equity considerations relevant to the trial; specialty experts; an implementation research expert; an expert tied to the specific intervention type, if it is unique; a disease-specific expert; and the number of experts in core disciplines such as biostatistics and pharmacokinetics/pharmacodynamics. |
| ML2 | Managed | Specific, repeated areas of needed expertise, or “expert roles”, have been identified, such as biostatisticians, epidemiologists, or pharmaco-kineticists. Attempts are made, but not always achieved, to deliver minimum “expertise coverage” within and across reviews. Experts have differences in agendas, amount of time to give to the review, number of prior reviews, or processes. Specialized or “custom-to-trial” expert needs identified as aspirational. There is consensus for what the minimum, core, required set of breadth of topical expertise is in a review panel. |
| ML1 | Initial | Most reviews do not have reviewers with experience in special or secondary trial elements such as disease, intervention type, site geography, and participant attributes. Representation of expertise for a particular trial element, or “expertise coverage” has sparsity within and across reviews and is not predictable. For example, there may be an odd time when a review panel includes no expert for biostatistics or for pharmacokinetics. There is no consensus on what the core, required set of breadth of expertise in reviewers is. |

Process Area 3, Depth of reviewer expertise

Terms included:

Depth: “In subject matter expertise, the ever-increasing ability to identify, characterize, and extemporize on a myriad number of discrete facts, concepts and ideas related to a topic area, using or creating interconnected networks of meaning and significance in the process.” [2] Here, depth of reviewer expertise relates to the skill of any single reviewer. An expert reviewer with a very high depth of expertise would be considered a known figure in their field, a recognized author, or sought after speaker in that niche. Breadth of review expertise, on the other hand, refers to the range covered by all reviewers on a review panel.

Topgrading: “Topgrading simply means proactively seeking out and employing the most talented people available, redeploying those of lesser ability…employing only A players [top talent].” [3]

**Table S3. Process Area 3, Depth of reviewer expertise**

| **Maturity Code** | **Maturity Levels** | **Capabilities, Depth of reviewer expertise** |
| --- | --- | --- |
| ML5 | Innovating | Automated or innovative methods for "topgrading" occur to continue to increase the level of expertise among those already with expert status. Artificial intelligence may be used to create "digital twins" of deep experts, and tested for superior review recommendations. Data science may be used to predict timelines of turnover among expert reviewers toward ensuring no gaps in coverage. Prediction of future new study types and domains identify new types of experts to be recruited in advance. Because of the pace of technology improvement and new inventions in the space, not all innovations can be adequately captured and described here. |
| ML4 | Predictable | Insights from historical review recommendations are used to predict optimal remedial or *de novo* education or experience to further deepen expertise. Analysis of review recommendations over time may identify reviewers who are outliers within expertise area-study type combinations, toward quality improvement. A 'bench' of future review capacity may be built, consisting of high-quality experts pre-trained in the review process, in order to not have a decline in review quality in situations of existing reviewer turnover. Unique reviewers may, during or between reviews, create original content, resources, data, or tools that reinforce their review feedback, and provide this content to the review team. |
| ML3 | Standardized | A minimum level of depth of expertise is set and practiced. Financial minimums, boundaries, resources, and methods are set to ensure the review process can afford deep experts. Multiple defined sources, registries or catalogs of experts are established. Standard recruiting and onboarding content or tools may be developed to ensure a high quality of new expert reviewers. Documentation exists to describe to reviewers what is expected of them, toward their understanding that particular parts of their expertise may or may not be needed, to further ensure they are spending their review bandwidth in their area of expertise. |
| ML2 | Managed | Approaches for recruiting expert reviewers are practiced and improved. Breadth of expertise in a review team is sacrificed if needed to ensure minimum depth of expertise standards. An understanding of current and future financial budgets for procuring depth of expertise, if necessary, are understood. At times, adding multiple experts in a domain who review in serial, the second reviewer using the recommendations of the first, may be used as a way to increase depth of expertise. Depth is not uniform across reviews. |
| ML1 | Initial | Based on the definition of 'expert' herein, some members of some review teams are experts, and some are not. In order to achieve breadth of expertise, some sacrifice in depth of expertise is made. Financial and time investments needed to “source and hire” deep experts may not be affordable yet. |

Process Area 4, Iterative

Terms included:

Tranche: One of the parts into which an investment is divided. In this case, the “investment” of reviewer panelists’ time is not done in a single sitting, with all reviewers critiquing simultaneously. Instead, a smaller review panel critiques together, and their feedback is fed to a second reviewer panel who does their critique (the second “tranche”). Multiple tranches of review, if managed well, may lead to the creation of a wider set of recommendation topics as well as enabling deeper considerations on initially-identified recommendations.

Reviewer panel: An assembled team of experts tasked to read and critique a clinical trial protocol and associated documents on behalf of a funder (where the team is not part of the PI group, nor has a conflict of interest), who makes their comments and recommendations around the same time, and often represents a wide range of technical competencies rather than all being expert in one discipline.

**Table S4. Process Area 4, Iterative**

| **Maturity Code** | **Maturity Levels** | **Capabilities, Iterative review** |
| --- | --- | --- |
| ML5 | Innovating | Artificial intelligence could analyze historical data to identify and cue optimal orders of communication, duration and type of communication, rounds of communication within and across stakeholder groups, to lead to feedback eliciting more protocol changes. Particular order of iterative review across reviewer roles could be optimized with higher order processing. For example, it may be that implementation research expertise must be used to identify possible study change recommendations first, then a biostatistician needs to review those recommendations to identify strength or risk before the whole recommendation is delivered, as opposed to biostatistical review first. Because of the pace of technology improvement and new inventions in the space, not all innovations can be adequately captured and described here. |
| ML4 | Predictable | Processes for inter-reviewer debate are streamlined and tuned based on prior experiential data. Management begins to predict optimal composition of later tranches/reviewer panels based on initial tranche/reviewer panel composition, especially by certain trial context. Identification of opportunities to add an early tranche of concept review during early, pre-protocol, conversational stages is sought. |
| ML3 | Standardized | The cadence across review teams for communication, clarification, document, and feedback delivery is well-established and maintained. There is standardization of role types of reviewers in the first, second, and further tranches. Schedules and processes for members of each tranche are standardized and communicated. Consistent achievement of no duplication of expertise in any one tranche, with minimal sparsity, is attained, for powering efficient iteration. For example, a human 'curator' who convenes meetings with each tranche of review, and otherwise removes duplicate comments and manages information flow (i.e., a “compiler”) may emerge. A culture of respect and equality is sought between different tranches of reviewers or reviewer subgroups. |
| ML2 | Managed | More than one tranche of review panel exists, deliberately. The internal team managing reviews encourages reviewers to consider and discuss recommendations or commentary from other reviewers within and across tranches, or have meetings to do such consideration directly. Availability of documents and information does not follow a standard timeline. There are no consistent means for the second tranche of reviewers to gain clarification on commentary from initial review tranche. Processes are not worked out on final decision-making power across reviewers. |
| ML1 | Initial | An individual reviewer's recommendations are often not visible to other reviewers. No iterative tranches of reviews with regard to communication with the PI: review comments go directly to PI from reviewer panel. Occasionally a cycling of comments will occur within a single tranche of review, and individual feedback is adjusted. |

Process Area 5, Information-enhanced

Terms included:

Information: “Here, “information-as-thing”, the attributive use of “information” to denote things regarded as informative; varieties include data, text, document, and objects that can be stored and retrieved—as opposed to processes or knowledge.” [4] Specifically, that when reviewers are enabled with precise, efficient, rich, and actionable information at the right time, there is a likelihood of a better review.

An information-enhanced review is one where reviewers have the benefit of additional, just-in-time, documents that are original and completed both by the PI—in the form of a fixed request for specific information beyond the protocol—and the reviewer organization—in the form of aggregated relevant contextual variables specific to the trial to be reviewed.

**Table S5. Process Area 5, Information-enhanced**

| **Maturity Code** | **Maturity Levels** | **Capabilities, Information-enhanced review** |
| --- | --- | --- |
| ML5 | Innovating | External data, both around similar studies and the review trial, are used to predict uninformativeness and made available to reviewers. External information, such as libraries of study protocols, are used to identify opportunities for review recommendations. Information is synthesized, either from past reviews, current recommendations, or other sources to help with individual reviews or an aggregate level of reviews. Data science is used to evaluate novel data, real-world data, and other information to identify if there are reliable associations with variables of interest, as well as associations between review feedback and informative outcomes in reviewed trials. Because of the pace of technology improvement and new inventions in the space, not all innovations can be adequately captured and described here. |
| ML4 | Predictable | Accompanying, enriching data, such as completed statistical analysis plans, target product profiles, target policy profiles, participant communication plans, dissemination plans, intermediate PI responses, or additional information requested by reviewers are collected and provided back to reviewers reliably. Protocol changes made are used to predict improved profiles of information compiled. Analytics on the effect or likelihood of uninformativeness associated with missing ancillary data, benchmarks, SAPs, and other information are performed. |
| ML3 | Standardized | Consistent and standard information requests for additional information beyond the protocol by reviewing organization, and delivery by the PI of the same, enable improved review recommendations and faster reviews. The review organization has designed a fixed template of required or requested information to be compiled by the PI just prior to the review, to bring further clarity to certain key aspects of the protocol. Prior reviews identify core information of reviewer interest that, when collected from similar, recent trials, becomes a ‘benchmark’ against which to validate the trial being reviewed; these benchmarks are created for and given to review panels. |
| ML2 | Managed | Trial protocols are often complete and usually arrive on time. Requests are made to the PI to provide new content outside of the protocol to aid reviewers; such requests are not always fulfilled. Financial or other resources may exist to support PI’s or reviewers compiling their own review-enriching information, within certain specified bounds. Definitions of minimum requirements of study protocols exist. Alternate types of reviews may be implemented for situations where minimum criteria of information in a review are not met, such as when only a draft study synopsis is available. |
| ML1 | Initial | Profiles of information that reviewers would like to receive along with a protocol are discussed and requested. Reviews sometimes include a complete study protocol, and sometimes rely only on a study synopsis or largely incomplete protocol. Rarely does enhancing or enriching information—either incrementally solicited from the PI at the time of the review, or developed by the funder specific to the review—accompany a protocol. |

Process Area 6, Solution-oriented

Terms included:

Solution: Implementation of a technique or method—enabled by adjustments by or with people, process, information, or investment—to improve capability or delivery in a situation. Here, a solution is a change in some attribute of the trial, trial design, or trial design method that increases the likelihood that the trial will end informatively, in the opinion of the reviewers.

While communication may be a solution in itself or part of a solution, communication is handled separately, as Process Area 8, “Collaborative reviews”.

**Table S6. Process Area 6, Solution-oriented**

| **Maturity Code** | **Maturity Levels** | **Capabilities, Solution-oriented review** |
| --- | --- | --- |
| ML5 | Innovating | Customization or optimization of review process to grantee and study type may occur, toward the review approach most likely to lead to a solution implementation for the review issues found. Opportunistic incremental funding for review recommendation-driven changes is routinized and data-driven. Incentives for grantees to adjust to informativeness recommendations may be tested. Understanding informativeness ‘returns’ tied to changes in protocols might occur. Optimization of aggressiveness of review recommendation or a mix of trial planning grant investments could be developed. Because of the pace of technology improvement and new inventions in the space, not all innovations can be adequately captured and described here. |
| ML4 | Predictable | Networks of subject matter experts (SME) are made available, and in some cases, funded to aid in solutions. Review recommendation metadata that is consistently tagged is used in analytics to help reviewers improve. If solutions to trial risks of uninformativeness come with financial support, such as trial planning grants, historical outcomes of same may be used to predict improvements in current solution-centric grants. Feasibility, cost, and appropriateness of solutions recommended are matched with trial attributes and become indicative and actionable. Variations in review recommended solutions for the same issues identified in reviews across time are identified. |
| ML3 | Standardized | Review feedback delivered in consistent format for every review, in a timeframe that is actionable. Feedback includes prescriptive or action-centric wording, often with an example solution. Types, sizes, and formats of follow-on assistance to grantees tied to feedback begin to take shape and be templatized or packaged. Other PI-focused 'solutions' may be aided, incentivized, or subsidized with trial planning grants or other support. Considerations for variation across trials or PI teams may be made (e.g., language translation for reviewers or PIs designing solutions & responses). |
| ML2 | Managed | There is some oversight and management of feedback, so it is not cryptic, sparse, or otherwise non-actionable. Feedback recommendations are translatable into solution-centric action by the PI. Review feedback likely to arrive sooner or within a timeframe that corrective action can be taken or attempted. In some cases, feedback can be packaged by PIs into requests for financial assistance to solve the design opportunity. The review recommendation suggestions presence, practicality, and detail may vary based on who is on the review panel, or who is compiling recommendations for the PI. |
| ML1 | Initial | Review feedback is delivered as insights or facts, and only occasionally is delivered in writing with recommended solutions with examples. Feedback is not proofread and edited, and therefore delivered in different 'voices', perhaps without context, further ensuring difficulty for PI in translating feedback to solution concepts. Recommended actions are often missing. No option for iterative dialogue with reviewers is offered, nor is question & answer available. |

**Table S7. Process Area 7, Software-enabled**

| **Maturity Code** | **Maturity Levels** | **Capabilities, Software-enabled review** |
| --- | --- | --- |
| ML5 | Innovating | Previously unleveraged software types are experimented with to increase review speed, efficiency, and quality. The need for additional language support may be identified and addressed. Additional and perhaps wider groups of stakeholders are addressed who could, through software, be enabled with transparency or other services. Alternate software-driven environments for reviews to take place, such as the metaverse, may be plausible. Because of the pace of technology improvement and new inventions in the space, not all innovations can be adequately captured and described here. |
| ML4 | Predictable | User feedback is collected by all stakeholder types on experiences with standard software tools used, and the results cue actions to improve the experience. The internal review managing team optimizes software used to reach improved level of predictability, speed, efficiency, and quality in all aspects. Service levels are established, including for software outages. Software-specific dictionaries, users guides, common definitions of elements, and metadata are established. Usage information, user complaints, and lack of scalability to new capacity building all are collected toward knowing when to move to different software. |
| ML3 | Standardized | An internal collaboration platform is used for those managing reviews and reviewers and for storing review documents. One software is selected for data curation and analytics. A software-centric solution may be rolled out to PIs being reviewed for document management and other communication. Software may be tested and settled on for specific needs of the review management team (e.g., project management or customer relationship management) or, in advanced cases, for the reviewers (e.g., statistical simulation or real-world evidence analysis of epidemiology data). Templates frozen and always used for recording and delivering review feedback and recommendations to PIs. Training and analytic software has been through a selection process, identified, and is in various stages of implementation. Cloud storage is likely in use. |
| ML2 | Managed | Software is used to track review statistics and review recommendations. Different software may be used across review teams simultaneously. Turnover in software tools happens over time as the most feasible tools are settled on. Privacy, confidentiality, and cybersecurity is ensured. Collaboration tools may be crude or sophisticated. Software is usually not cloud-based. Templates experimented with for delivering review feedback and recommendations. |
| ML1 | Initial | No enterprise-class or collaborative software is used in some of these reviews. Widespread, standard, but single user-centric software such as Microsoft Word or Microsoft Excel may be used frequently. Version control or lost data issues can occur. Some reviews include all recommendations tracked and stored historically. |

Process Area 8, Collaborative

Terms included:

Collaboration: “A process where key stakeholders constructively explore different aspects of a problem domain and search for solutions, leading to joint decision making about the potential future of that domain, while using agreed-upon rules and a temporary structure.” [5]

There are limits to collaboration; limitations could be legal, organizational, and functional. In fact, one attribute of success in a review system might be an “arm’s length” distance or element of independence between reviewers and authors of a protocol. What seems clear is that *outside* the areas where independence and reserve are required, increased collaboration makes the review better.

**Table S8. Process Area 8, Collaborative**

| **Maturity Code** | **Maturity Levels** | **Capabilities, Collaborative review** |
| --- | --- | --- |
| ML5 | Innovating | New collaboration technologies and software are piloted for potential addition to the program. Deeper slicing and dicing of recommendation uptake data tied to communication and collaboration information occurs and is acted upon. New types of communication and collaboration are tested. "Joint reviews" with external stakeholder reviewers are enabled. Because of the pace of technology improvement and new inventions in the space, not all innovations can be adequately captured and described here. |
| ML4 | Predictable | The most productive approaches to the review process are identified through data analysis of uptake of review recommendations. Collaboration, where appropriate, begins to be customized based on PI attributes. Uptake of change management, communication-centric process improvement or implementation programs happens, such as the AIM Change Management Methodology [6], to aid in successful collaboration. Collection of data on collaboration effectiveness, and use of that data, occurs. As collaboration moderates protocol changes, insights on collaboration are driven into other process areas. Collaboration learnings drive choices in enhancing software, information, and other program areas. |
| ML3 | Standardized | Communication of schedules and milestone activities is proactively delivered in standard ways across all review stakeholders. Human collaboration “curators” or relationship managers facilitate easier engagement amongst parties. Service levels for communication exist. Reviewers see others’ comments and have collaboration mechanisms and defined time-based cues to collaborate. Standards for collaboration and communication between reviewers and PIs are established and seen as an important facet of collaboration. Collaboration with non-reviewer, non-PI stakeholders is defined, if any exist. Reviewers speak to PIs after delivering their written recommendations and receiving PI’s written responses to achieve maximum possible collaboration. |
| ML2 | Managed | Communication from the internal team managing reviews to reviewers and grantees is ensured, if inconsistent or delayed at times. Communication between stakeholders is routed through the hub of an internal team managing the review process. Episodes of routine, repeating communication are identified, and best practices are identified. Collaboration among reviewers is specified and prompted. Some reviews include reviewer-to-reviewer collaboration and reviewer-to-PI collaboration. |
| ML1 | Initial | Collaboration between the internal team managing the review process and grantees may be sporadic, irregular, and not proactive. Reviewers will generally not be collaborating or even communicating with each other, nor with grantees. Sporadic bursts of collaboration may occur. |

Process Area 9, Rich in data & analytics

Terms included:

Visualization: “Data visualization are based on qualitative or quantitative data and result in an image, graphical depiction, or chart, that is representative of the raw data, readable by viewers and supports exploration, examination, and communication of the data.” [7]

**Table S9. Process Area 9, Rich in data & analytics**

| **Maturity Code** | **Maturity Levels** | **Capabilities, review rich in data & analytics** |
| --- | --- | --- |
| ML5 | Innovating | Artificial intelligence is used to make predictions about future results in all areas of the review process. Report libraries are documented with service levels for publish/subscribe and metadata for all user types. Opportunities for bespoke analytics and visualizations are offered to executives with dedicated reporting experts available to deliver same. Fast turnaround is enabled for situations when executive decision-making has need of actionable intelligence. Data sharing with peer partner organizations may occur. Because of the pace of technology improvement and new inventions in the space, not all innovations can be adequately captured and described here. |
| ML4 | Predictable | External or novel data may be added to the internal trial and review data to enrich potential analytics. Information on review mechanics (personnel involved, multiple types of dates during the process, durations, and other operational facts) are used to create actionable insights. Information about trends in recommendations and outliers are provided to decision makers and reviewers. A wider set of stakeholders are, in an automated way, given access to visualizations. Historical data is used to drive improvements regularly (e.g., every quarter). |
| ML3 | Standardized | A wider set of data is collected in a commercial off the shelf software, using common definitions. No documented data dictionary exists. Multiple stakeholders have been able to request specific data fields be captured in the future. A regular yet limited set of visualizations is produced that includes some review metadata. It may take some time to collect, curate, and cleanse data, but these processes are routinized. These analytics are not offered to all parties; they include tables and graphs. Most questions answered relate to 'slicing and dicing' data on the output—often counts—of reviews in aggregate. Answering the question “What happened…” about a review from the distant past is possible without using an oral tradition. |
| ML2 | Managed | A core set of trial elements, trial attributes, review components, and review metadata are all defined as being worthy of capture. Experimentation with data formats and approaches for capture and storage are made. Data sparsity exists. Depth of data varies. Formats are not consistent. Storage is in a mix of locations. Custom visualizations might be created. |
| ML1 | Initial | Some data is captured during and after the review, in different formats. No standard definitions are set for trial attributes^[[1]](#footnote-1)^ or for review metadata^[[2]](#footnote-2)^ that will be stored. Review comments or recommendations are usually recorded as they are delivered. No visualizations are offered to any stakeholder. Questions are not solicited from stakeholders that would require analysis of historical review metadata or attributes. |

Process Area 10, Reliability and quality^[[3]](#footnote-3)^

Terms included:

Reliability Growth: “Reliability growth is defined as the positive improvement in a reliability metric or parameter of a product (e.g., a system) over a period of time due to changes in the product's design and/or the manufacturing process.” [8]

Quality Control: “While reliability is concerned with the performance of a product over its lifetime, quality control is concerned with performance of a product at a point in time.”[9]

**Table S10. Process Area 10, Reliability and quality**

| **Maturity Code** | **Maturity Levels** | **Capabilities, Reliability and quality** |
| --- | --- | --- |
| ML5 | Innovating | Methods for increasingly frequent monitoring and measurement of the program ‘system’ are experimented with, including the use of Bayesian statistical approaches. Attempts could be made to apply external quality improvement through unified “mash-up” reviews with other panels currently reviewing the same trial. Novel applications of expert opinion, the Dempster-Shafer theory, or fuzzy rules could be attempted [10]. Because of the pace of technology improvement and new inventions, not all innovations can be adequately captured and described here. |
| ML4 | Predictable | Processes are documented for handling complaints, so that the information makes its way to program systems, definitions, and mechanics toward increasing reliability growth. Over time, an increasing frequency occurs of assessing data on review quality and review impact, and driving those assessment to review practitioners and into system improvements. Multiple definitions of review impact are documented, tied to single and multiple groups of stakeholders, and assessments and efforts for reliability growth and quality are made. |
| ML3 | Standardized | A level of minimum quality guides delivery of all reviews. Failures are identified quickly, stakeholders are notified, and the situation is remediated. Costs per review occur within tight or predictable bounds, and growth or decreases in costs are predicted. All program members have defined and maintained and continuing minimum training in program-specific knowledge required, such as change management programs and domain knowledge (e.g., informativeness). Routine enhancements for reliability growth or quality that are suggested, tested, and implemented are defined and documented. Defined frequency of data collection, review tests, surveys, and other techniques to measure reliability and quality will be standardized. Reliability is high enough to “clone” a review team: insert a new independent, separate review team and onboard them to perform quality reviews alongside an incumbent team. |
| ML2 | Managed | The attributes of successful, acceptable, and failed reviews are defined, agreed, and documented, but no tests or scoring are applied systematically. Data is collected, perhaps arbitrarily, post-review, from various stakeholders, toward improved information about reliability and quality. Leadership, communication, and consistency aim to routinize the techniques to deliver high-availability and high-maintainability reviews. Minimum and maximum per-review cost amounts are set and tracked. Implementation-wise, scenarios are identified that strain quality, reliability, cost, and other key factors. Change management, data systems, domain knowledge (e.g., informativeness), and other resources that benefit members are applied inconsistently. |
| ML1 | Initial | “The first prototypes produced during the development of a new complex system will contain design, manufacturing and/or engineering deficiencies.” [6] The concept of a “quality review” is being defined by program members or management, but is not set. Reaching consensus on whether a ‘failed’ review has occurred is difficult. Techniques have been identified to deliver reviews which include reasonable or high availability of a review to a client, customer, or stakeholder. Techniques for maintaining consistency are envisioned. Techniques for addressing change management challenges are experimented with. |

**Table S11. Process Area 11, Time appropriate**

| **Maturity Code** | **Maturity Levels** | **Capabilities, Time appropriate** |
| --- | --- | --- |
| ML5 | Innovating | Multiple stakeholders are solicited about the utility of fast reviews, and whether they are appropriate and valuable. Feedback continues to be collected about the verity of a PI or other stakeholder needing a review completed ‘immediately’. Information is collected or estimated regarding cost/benefit of faster reviews relative to loss of informativeness due to lower quality recommendations. Faster review times are attempted or assessed by techniques such as pre-staging packaged recommendations by disease-trial phase combination, or by utilizing checklists for expert reviewers to avoid free-flowing protocol interpretation. Methods for activating internal staff and expert reviewers in emergencies that decrease burnout are tested. |
| ML4 | Predictable | Data is collected about review and review stage durations systematically. That data is analyzed statistically to identify causal factors of what drives time overages. If data is available on review quality, it can be an input to the trade-off between duration and quality. Routinized or automated communication is delivered to reviewers, PIs, or other stakeholders when they are approaching or exceeding deadlines. Remediation is applied for reviewers who repeatedly cannot deliver on time. Identification early of PIs who likely will not be able to deliver documents on time or will try and change or delay information-exchange meetings is performed. Steps are taken to proactively address these issues. Management of external or contextual factors that decrease predictability are addressed. |
| ML3 | Standardized | The time extent of the review—the duration—has tight boundaries. Expectations are high that the boundaries of review duration are not exceeded. While causal factors of reviews going overtime are not collected systematically and statistically evaluated, work is applied to gut feel sensibilities of what makes reviews go ‘long’. Documented guidelines of who and what is included in each review stage, and how long it can take, are provided in advance per review, as well as in reviewer onboarding. Flexibility, in response to unexpected events, is bounded. Durations are measured in real-time, and communication is high. Repeated patterns of contextual variation may lead to multiple review offerings with different durations. (e.g., expedited and routine). |
| ML2 | Managed | Stages of reviews are identified and defined. Estimates of review duration (e.g., minimum, maximum, median, and mean durations) are considered for the entire review but not the stages within a review span. A single review’s scope or definition of output is defined and linked to a typical duration; these expectations are shared with PIs and other stakeholders. Attempts are made to not let reviews exceed a certain number of weeks. It is more likely that reviews go long due to exigencies than because of trial-specific variation. Fixed time “policies” are offered regardless of pressures from certain stakeholders, however exceptions are often made to these time-centric policies. |
| ML1 | Initial | Duration of reviews cannot be predicted. Variation across reviews in duration is high or meaningful. Reviews requiring speed invariably have loss of review quality that could include lower quality trial recommendations, less engagement with PIs, less collaboration, or less focus on informativeness. |

**SUPPLEMENTARY REFERENCES**

1. Zarin DA, Goodman SN, Kimmelman J. Harms from uninformative clinical trials. Jama. 2019 Sep 3;322(9):813-4. doi:10.1001/jama.2019.9892
2. Wineburg S. Beyond “breadth and depth”: Subject matter knowledge and assessment. Theory into practice. 1997 Sep 1;36(4):255-61. doi.org/10.1080/00405849709543776
3. Smart BD, Smart GH. Topgrading the organization. Directors & Boards. 1997 Mar 22;21:22-9.
4. Buckland MK. Information as thing. Journal of the American Society for information science. 1991 Jun;42(5):351-60. DOI:10.1002/(sici)1097-4571(199106)42:5<351::aid-asi5>3.0.co;2-3
5. Wood DJ, Gray B. Toward a comprehensive theory of collaboration. The Journal of applied behavioral science. 1991 Jun;27(2):139-62. doi.org/10.1177/0021886391272001
6. The AIM Change Management Methodology. Implementation Management Associates website. Accessed April 10, 2022. https://www.imaworldwide.com/aim-change-management-methodology
7. Azzam T, Evergreen S, Germuth AA, Kistler SJ. Data visualization and evaluation. New Directions for Evaluation. 2013 Sep;2013(139):7-32. doi.org/10.1002/ev.20065
8. RGA Overview. ReliaWiki website. Last updated December 11, 2015. Accessed May 14, 2022. <https://www.reliawiki.com/index.php/RGA_Overview>
9. Introduction to Life Data Analysis. ReliaWiki website. Last updated December 16, 2015. Accessed May 14, 2022. <https://www.reliawiki.com/index.php/Introduction_to_Life_Data_Analysis>
10. Gupta G, Ghasemian H, Janvekar AA. A novel failure mode effect and criticality analysis (FMECA) using fuzzy rule-based method: A case study of industrial centrifugal pump. Engineering Failure Analysis. 2021 May 1;123:105305. doi.org/10.1016/j.engfailanal.2021.105305

1. Attributes are details, characteristics, features, or tags describing some unique facet or quality. Here, trial attributes might include the trial phase, trial site location(s), cost to the funder, duration, or sample size. [↑](#footnote-ref-1)
2. Metadata are “data about data”, and in this case refer to statistics or detail about the data collected about reviews. These might be the date review documents were delivered to reviewers, the number of reviewers in each review panel tranche, how many meetings occurred with the PI, or a ‘success store’ poll result from reviewers. [↑](#footnote-ref-2)
3. If an organization lacks resources sufficient to promptly attend to the Reliability and Quality process area, a best practice would be for them to document the process area as “deferred”, accompanied by appropriate review and approval. The hazard analysis or “failure mode, effects, and criticality analysis” records for their organization’s program would show that the reliability risk is not currently mitigated. [↑](#footnote-ref-3)
